# Improving the echocardiographic prediction of systolic anterior motion risk following mitral repair

**DOI:** 10.1186/1749-8090-8-S2-O2

**Published:** 2013-10-23

**Authors:** B Popa, G Musica, G Cerin, L Zamfir, M Diena

**Affiliations:** 1 Cardiac Ultrasonography Dpt, San Gaudenzio Clinic, Novara, Italy; 2 Cardiac Surgery Dpt, San Gaudenzio Clinic, Novara, Italy; 3 Cardiology Dpt, San Gaudenzio Clinic, Novara, Italy

## Background

One of the possible complications following the mitral valve repair is the systolic anterior motion (SAM) of the anterior mitral leaflet. The preoperative echocardiographic examination is essential for the identification of the patients at risk for this situation. The most frequently used indicators are the ratio between the AML and PML (AP ratio) and the distance from the coaptation line to the interventricular septum (C-Sep). When repair of significantly redundant valves is performed additional parameters for SAM risk evaluation might be needed.

Objective was to investigate if the length of the mitral leaflets might be considered an additional parameter for the preoperative estimation of SAM risk.

## Methods

From March 2008 till December 2011, 159 patients (105 male) underwent complex mitral valve repair for degenerative mitral regurgitation. The SAM risk was evaluated using the classically described AP ratio (when smaller than 1.3) and the C-Sep (when smaller than 25mm); in addition a third parameter was introduced; namely the mitral leaflet length (when longer than 35mm).

## Results

The prevalence of the postoperative SAM following complex MV repair was 0,012 (two out of 159 pts) which is much lower than the published date over this situation (from about 2 to 16%).

## Conclusion

The length of the mitral valve leaflets is a valid adjunctive parameter for the prediction and avoidance of postoperative SAM. This information is fundamental for the surgical team as it provides a correct surgical planning.

